# Experiment-Based Interventions to Diabetic Retinopathy: Present and Advances

**DOI:** 10.3390/ijms23137005

**Published:** 2022-06-23

**Authors:** Siwei Liu, Yahan Ju, Ping Gu

**Affiliations:** 1 Department of Ophthalmology, Ninth People’s Hospital, Shanghai Jiao Tong University School of Medicine, Shanghai 200011, China; liusiwei000@sjtu.edu.cn (S.L.); juyahan731@sjtu.edu.cn (Y.J.); 2 Shanghai Key Laboratory of Orbital Diseases and Ocular Oncology, Shanghai 200011, China

**Keywords:** diabetic retinopathy, therapeutic strategy, stem cell therapy, genetic therapy

## Abstract

Diabetic retinopathy is the major blinding disease among working-age populations, which is becoming more significant due to the growth of diabetes. The metabolic-induced oxidative and inflammatory stress leads to the insult of neovascular unit, resulting in the core pathophysiology of diabetic retinopathy. Existing therapies focus on the inflammation, oxidation, and angiogenesis phenomena of diabetic retinopathy, without effect to radically cure the disease. This review also summarizes novel therapeutic attempts for diabetic retinopathy along with their advantages and disadvantages, mainly focusing on those using cellular and genetic techniques to achieve remission on a fundamental level of disease.

## 1. Introduction

Diabetic retinopathy (DR), a common complication of diabetes mellitus, is the leading cause of blindness among middle-aged and elderly populations, which is presented in about 20% of diabetic patients [1]. The lifetime risk to develop DR in type 2 diabetes mellitus (T2DM) patients is 50–60% and 90% in patients with type 1 diabetes mellitus (T1DM) [2].

According to neovascularization conditions, DR can be classified into non-proliferative DR (NPDR) or proliferative DR (PDR). Diabetic patients with retinopathy may present without significant clinical manifestations. In the early stages of DR, even before microvascular changes can be visible in ophthalmological examinations, retinal damages such as neural apoptosis and reactive gliosis can occur, resulting in thinning of the retinal nerve fiber layer and ganglion cell layer. As the disease progresses, vascular histopathological changes such as basement membrane thickening, acellular capillaries and pericyte loss can be observed [3]. The occurrence of neovascularization indicates the progression of DR to its end-stage PDR. These new vessels are prone to rupture, owing to their fragile architecture and tendency to grow into a vitreous body, which will then lead to fibrotic contraction and cause tractional retinal detachment, manifesting as severe vision loss in late stage of DR [4].

During the progression of DR, pathological processes including metabolic dysregulations, oxidative stress, and inflammation are playing significant roles, altering the neurovascular functions of the retina. Nowadays, interventions of DR include preventive strategies and interventions including corticosteroids, anti-vascular endothelial growth factor (VEGF) agents, laser photocoagulation, surgeries, etc. However, these strategies rarely reverse the radial pathological changes of the diabetic retina, let along the side effects of invasive surgeries and repeated injections, leading to poor long-term prognosis of DR. Therefore, novel treatments, mainly based on cellular and genetic interventions, aiming at achieving long-term and effective disease reverse are continuously under investigation, offering new hope for DR treatment. Herein, we will briefly introduce the pathophysiology of DR and current treatment options and revise the experiment-based novel strategies for DR with future prospects.

## 2. Pathophysiology of DR

### 2.1. Metabolism, Oxidative Stress, and Inflammation

**Figure 1:**
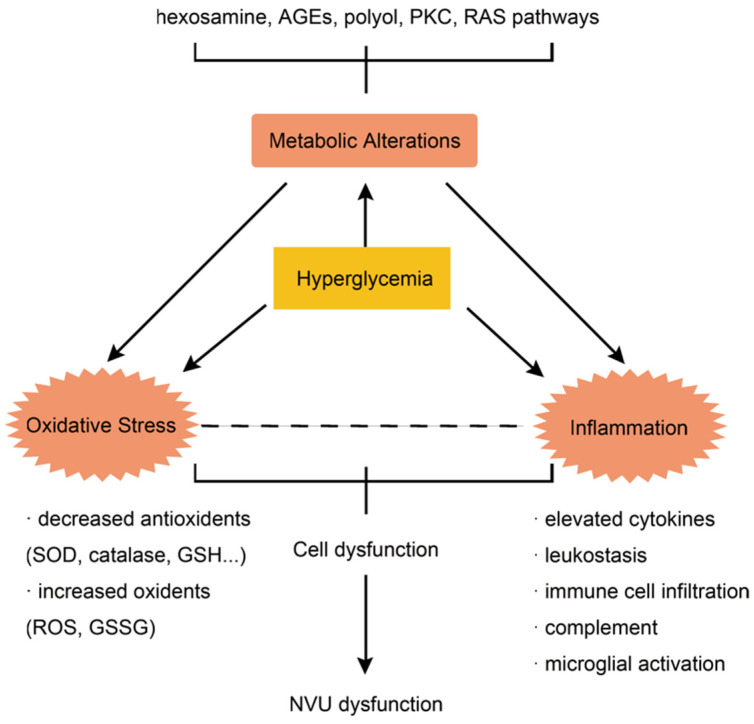
The interaction of metabolic dysregulation, inflammation and oxidative stress underlies the pathophysiology of DR.

As a complication of diabetes mellitus, hyperglycemia is one of the essential contributions to DR development. Several underlying mechanisms of how elevated blood glucose leads to DR are identified, including the hexosamine pathway, advanced glycation end products accumulation, polyol pathway, protein kinase C pathway and poly polymerase activation [5]. These pathways drive metabolic dysfunctions, resulting in further insults, causing DR formation and progression. Evidence has also shown the effect of renin angiotensin system (RAS) activation in DR and its potential as a therapeutic target, leading to multiple trials of medications such as losartan, candesartan and enalapril on DR intervention [6,7,8]. The metabolic dysregulations, together with oxidative and inflammatory imbalance, lead to the pathological presentation of DR (Figure 1).

The hyperglycemia-induced pathways above can all lead to excessive oxidative stress, which is another key aspect of DR formation. The overproduction of oxidative stress is driven by the production of superoxide from sources such as electron transport chain and cytochrome P450 [9]. Owing to its high content of polyunsaturated fatty acids, oxygen demand and glucose oxidation, the retina is highly sensitive to oxidative stress. Hyperglycemia can lead to excessive oxidative stress, which will inevitably insult the vulnerable retina, resulting in retinal complications. Increased reactive oxygen species (ROS) are detected in retina, as well as elevated membrane lipid peroxidation and DNA oxidative damages [10]. Oxidative stress and the destruction of redox homeostasis are recognized as the “unifying mechanism” of DR, as well as the metabolic memory phenomenon, which refers to the persistence of retinal insults even when blood glucose is well controlled [11]. The accumulation of ROS is thought to be one of the major drivers of the persistence of retinal insults, as explained in the pre-mentioned metabolic memory phenomenon. Furthermore, as oxidative stress is caused by an imbalance between oxidants and antioxidants, antioxidants such as superoxide dismutase, catalase, glutathione reductase, glutathione peroxidase, and glutathione are all diminished in retina in the case of diabetes, which in turn aggravates hyperglycemia-induced oxidative stress [12,13]. The imbalance of the redox system can cause a dysregulation of inflammation and vascularization, leading to microvascular dysfunction and neurodegeneration in DR [14].

Inflammation is also a critical driver of DR development and progression, which is intrinsically related to metabolic dysregulations and excessive oxidative stress. Inflammatory features such as elevated cytokines, leukostasis, complement and microglial activation are detected in patients with DR [15]. The elevation of cytokines produced by activated microglia, endothelial cells, and even neurons represents the participation of inflammatory responses of all layers of the retina in the pathogenesis of DR [15]. Some of the inflammatory processes are thought to cause an early insult of neuronal cells in the retina of diabetic patients [16]. Inflammation is also documented as a key component of DR pathogenesis via capillary damage and hypoxia induction, leading to increased VEGF expression and neovascularization [17].

### 2.2. Neurodegeneration and Neurovascular Unit

Microvasculopathy alone cannot explain the early loss of retinal function and peripheral nerve involvement in DR patients. Therefore, neurodegeneration is gradually viewed as part of the pathophysiology of DR. Evidence has shown that retinal ganglion cells and amacrine cells are the earliest neurons among which apoptosis is detected, whereas the apoptotic rate of photoreceptors is increased as well [5,18]. As vascular and neural impairments both present in DR pathology, the concept neurovascular unit (NVU) was put forward to combine all the theories above together. It is first used to describe the blood–brain barrier and then applied to the retina to study conditions such as DR [18]. The concept of NVU views retina as a whole functional unit including neural, vascular, and supporting cells, in which all components can communicate to each other and maintain the integrity of the blood–retinal barrier. Autoregulation is one of the most important physiological roles of NVU to maintain normal visual function via matching retinal blood flow with changes in metabolic activity. This so-called “functional hyperemia” phenomenon, presenting as flicker–evoker vasodilation, is essential for the sensitive retina to survive in the variating environment [19]. Such a phenomenon is found impaired in asymptomatic early-stage DR patients, suggesting that the dysregulation of NVU is possibly central to DR pathogenesis [20].

## 3. Advances on Current Treatment and Limitations

### 3.1. Prevention

As metabolic disorder underlies the central etiology of DR, the controlling of blood glucose, blood pressure, and lipid levels are viewed as the most basic aspects of DR prevention and interventions. Evidence has proved that tight control of glycemia with glycated hemoglobin level below 7% can significantly reduce the development and progression of DR in diabetic populations [21]. In patients with both diabetes and hypertension, blood pressure control is also proved to be effective in reducing the risk of DR progression. A study demonstrated that tighter control of blood pressure, with a target below 150/85 mmHg versus 180/105 mmHg, reduced DR progression risk by one-third, with a reduction of vision loss by 50% in T2DM [22]. However, it did not show a lasting effect without long-term blood pressure maintenance in contrast to glycemic control [23]. Dyslipidemia, as a significant aspect of metabolic dysregulations, has also been found to increase the incidence and severity of DR [24].

### 3.2. Anti-VEGF Agents

Medications targeting angiogenesis are commonly used for severe-stage DR treatment, as neovascularization is the crucial pathway of the development of PDR and diabetic macular edema (DME). One of the most thoroughly studied targets is VEGF. Drugs such as bevacizumab, aflibercept, and ranibizumab are investigated in clinical trials for DR intervention and showed positive results, especially for DME and PDR [25,26,27,28,29]. Although most studies turned out with no significant safety issue, it should be concerned that long-term repeated anti-VEGF injections may cause neurodegeneration of the retina and impact choriocapillaris [30]. The potential side effects caused by the systematic distribution of anti-VEGF medications cannot be ignored as well [31]. Anti-VEGF therapy has the potential to increase cardiovascular risk rate, leading to rare but severe events such as myocardial infarction or stroke [32]. Furthermore, researchers also found that suppression of VEGF level can lead to increased connective tissue growth factor (CTGF), which has the potential to develop severe complications such as tractional retinal detachment [33]. However, trials estimating the application of anti-VEGF agents in patients with diabetic retinopathy, as well as other ocular issues including age-related macular degeneration (AMD) and retinal vein occlusion, are not powered enough to correctly estimate the risk of these rare incidents [31,34]. Therefore, continued data from real-world surveillance are essential to support the routine use of anti-VEGF agents. 

### 3.3. Anti-Inflammatory Agents

Intraocular steroids are traditional interventions for DR patients, especially for those with DME and neovascularization [35]. Corticosteroids act by downregulating various inflammatory pathways and VEGF expression to suppress the pathological inflammation and neovascularization in DR [35]. However, repeated intraocular injections can cause considerable side effects such as infection, which limit their clinical use [2]. Corticosteroids such as dexamethasone are also proved to be effective for DME, with limited side effects of multiple injections of its implants, which is confirmed by a meta-analysis [36].

### 3.4. Other Interventions

In addition to the medications mentioned above, invasive interventions of DR including laser coagulation and vitrectomy are also key components of DR treatment. Although the exact mechanism underlying laser coagulation is not clear yet, it is commonly believed that coagulation will cause hemodynamic changes, which will relieve the hypoxia status of the remaining retina, thus preserving eye functions [37]. In patients with advanced disease not responding to available non-surgical interventions, with persistent hemorrhage, or with tractional retinal detachment, vitrectomy is required to rescue the remaining vision [2]. However, as invasive therapies, the side effects of laser coagulation and surgery cannot be ignored. In addition, such interventions cannot reverse the lost vision and the ultimate blindness destiny of DR patients, offering little hope to restore vision. Therefore, novel resolutions focusing on basic-level pathophysiological reversions are undergoing investigations.

## 4. Novel Experiment-Based Interventive Strategies for DR

**Scheme 1:**
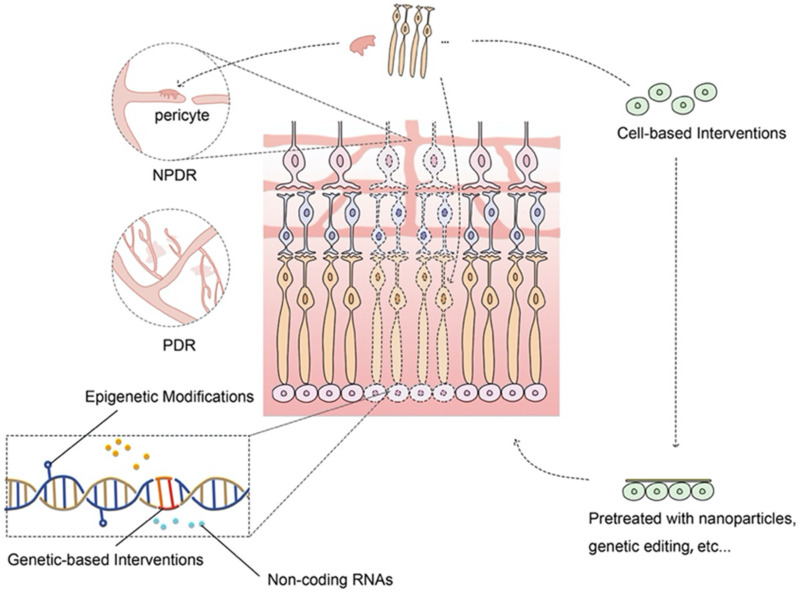
Novel experiment-based interventive strategies for DR. Novel strategies including cellular and genetic-based interventions can target the fundamental pathophysiological changes of DR, offering promising alternatives to improve outcomes of DR patients.

The existing treatments cannot reverse the primary problems of DR let alone the existence of adverse effects, resistance, and tolerance of traditional interventions. Emerging evidence has shown that novel therapeutic strategies including cell-based and genetic-based therapies which target the more radical levels of DR pathophysiology can offer a practical effect on DR as alternative approaches (Scheme 1).

### 4.1. Cell-Based Therapy for DR

#### 4.1.1. Cell-Based Therapy for NPDR

Due to the existence of cellular apoptosis and dysfunction in the progression of NPDR, stem cell therapies for cell replacement are being intensely studied as a potential therapeutic method for NPDR patients. Mesenchymal stromal cells (MSCs), endothelial progenitor cells (EPCs), induced pluripotent stem cells (iPSCs) and other types of stem cells have all been testified as potential cell replacement therapies for NPDR patients. MSCs including adipose stem cells (ASCs) and bone marrow-derived mesenchymal stem cells (BM-MSCs) are cell types that are most thoroughly studied to treat early-stage DR in rodent models. 

##### ASCs

Researchers have found that ASCs can functionally improve the pathological phenotype of DR induced by streptozotocin (STZ), presenting as significantly improved “b” wave amplitude measured by electroretinogram (ERG) [38,39]. In vivo studies have found that the administration of ASCs can significantly decrease retinal vascular apoptosis and leakage, prevent retinal ganglion cell loss, and improve BRB integrity in NPDR rodents, which further confirmed the therapeutic potency of ASCs on NPDR [38,40,41,42]. ASCs demonstrate a protective effect on NPDR mainly via two mechanisms: directly differentiating into retinal-related cells such as neural or perivascular-like cells, or reversing adverse microenvironment such as oxidative stress or inflammation. 

Studies have found that ASCs can differentiate into pericytes and participate in the fixation of early vasculopathy. In vitro studies show that ASCs can express pericyte-specific markers [38,43,44]. In vivo tests further confirmed that ASCs can migrate and integrate with retinal vasculature, adopting the typical pericyte morphological and functional behaviors [38,43]. Evidence also indicates that transforming growth factor beta (TGF-β1) can further enhance the pericyte phenotype of the administrated ASCs [45]. Knockdown of the platelet-derived growth factor receptor β (PDGFR-β) CD140b signaling pathway in ASCs, causing them less similar to pericytes, will subsequently reduce their protective effect on retinal endothelial cells [46]. Other researchers also found that ASCs have the potential to differentiate into photoreceptor and glial-like cells with the ability to repair the insulted NVU in DR, but whether ASCs are capable of differentiating into other retinal cell types is still controversial [40,41]. 

Surprisingly, the paracrine factors and extracellular vesicles secreted by ASCs are also effective for early DR, suggesting that alteration of microenvironment by ASCs is also an essential mechanism behind ASCs’ therapeutic effect. After ASCs intervention, intraocular levels of neurotrophic factors such as nerve growth factor (NGF), basic fibroblast growth factor and glial cell line-derived neurotrophic factor are found increased, and reduced oxidative stress and inflammatory factors can also be detected [38,40,47,48]. Rodents treated with hyperglycemia conditioned medium from ASCs (ASC-Cme) can also present with similar changes with those treated with ASCs [43,47]. One study comparing ASC-Cme with ASCs even suggests that ASC-Cme may have a better therapeutic effect on NPDR with a better functional improvement of the ASC-Cme group [47]. 

As a potential treatment for diabetes complication, ASCs displayed stable phenotype and proliferation in a high-glucose environment in multiple in vitro studies. Compared with human retinal pericytes, hyperglycemia has no impact on the adhesion and proliferation of ASCs, indicating them as favorable treatment options for DR patients [38,44,49]. Pro-angiogenic factors are found to be secreted by ASCs, which can be beneficial in reforming healthy and intact vasculatures in early DR. However, due to the presence of such pro-angiogenic effects, the application of ASCs on patients with neovascularization, such as PDR patients, should be carefully evaluated [42,49].

##### BM-MSCs

BM-MSCs are another potential option for cell-based NPDR treatment due to their plasticity and vascular-repairing effect [50]. BM-MSCs refer to a group of stem cells retrieved from bone marrow with mesenchymal differential potency. Researchers have detected a protective effect on DR rodents using different subtypes of BM-MSCs such as CD34+, CD14+ and CD133+ stem cells due to the heterogenous nature of BM-MSCs [51,52,53,54]. Intravitreally injected BM-MSCs are found to benefit visual function by improving ERG in STZ-induced rats [55]. Some studies have discovered that BM-MSCs can benefit NPDR via directly differentiating into functioning retinal structures such as photoreceptors and glial cells [55,56]. However, the engraftment ability and viability of BM-MSCs transplantation are controversial due to their heterogenous nature [55,57]. BM-MSCs can not only differentiate into retinal cells but also activate existing cell potential to treat retinopathy. Researchers have found that BM-MSCs are able to activate progenitor potential of retina müller glial cells (MGCs) [58]. Another way for BM-MSCs to treat NPDR is via secreting protective particles, such as brain-derived neurotrophic factors (BDNF), NGF and exosomes [52,58,59]. BM-MSCs can secret BDNF and increase the retinal BDNF level in STZ-induced mice in order to improve retinal cell survival [52]. BM-MSC-derived exosomes are another research target recently to investigate the effect of BM-MSCs on DR. The up-regulation of microRNA-486-3p is found to be induced by BM-MSCs exosomes via Toll-like receptor 4/nuclear factor-kappa-B axis repression in STZ-treated mice. Oxidative stress, inflammation, angiogenesis and cell apoptosis are all related to exosomes’ effect on high glucose treated MGCs [59]. However, a long-term safety study has questioned the clinical appliance of BM-MSCs. This study using Royal College of Surgeon rats, an animal model of AMD, demonstrated that BM-MSCs have the potential to circumvent BRB and migrate into non-target tissues after administration, which is a crucial side effect for researchers to overcome [60].

A subtype of BM-MSCs, bone marrow CD34+ cells, including endothelial progenitor cells (EPCs) are thought to be favorable cells for regenerative therapy due to their potential to regenerate damaged endothelium. Adult CD34+ cells are heterogeneous that contain EPCs and hematopoietic stem cells. Intravitreally injection of human CD34+ stem cells showed significantly increased vascular length and density in the superficial retinal capillary plexus, with no significant effect on deeper retinal vasculature in pre-clinical surveys [53]. These cells present their therapeutic benefits on retinopathy primarily via paracrine mechanisms, secreting proangiogenic factors and neurotrophic cytokines [61,62,63]. Targeting a multicellular involving disorder such as NPDR, CD34+ cells with paracrine nature are especially favorable due to the broad cellular effect. However, autologous circulating CD34+ stem cells are of limited use due to the impairment of high glucose stress [54,64,65]. Researchers are trying to overcome this barrier by using pre-treated cells for transplantation. One recent study using lentivirus vectors encoding IL-10 edited EPCs in treating NPDR rats showed decreased inflammation and significantly improved retinal vascular repair [66]. These augments of therapeutic effect may be potential methods to cope with the drawbacks of autologous transplantation. 

##### Other Stem Cells

Other cell types such as human umbilical cord mesenchymal stem cells (UC-MSCs) and iPSCs are also found to have potential therapeutic effects on NPDR. UC-MSCs are capable of differentiating into neural stem cells, increasing the number of surviving retinal ganglion cells, secreting neuroprotective factors and therefore treating NPDR in STZ and axotomized rats [67,68]. The decrease in BDNF in diabetic rats can also be prevented by neural stem cell transplantation [68]. Progenitor cells derived from iPSCs showed positive therapeutic effects in ischemic retinopathy models [69,70]. However, more in depth studies are required to apply these cells in the regenerative treatment of NPDR. 

#### 4.1.2. Cell-Based Therapy for PDR

Stem cells are of limited use in PDR treatment due to their proliferative nature to amplify neovascularization, which is the central manifestation of PDR. However, several studies still found evidence to support the use of cell-based interventions in PDR treatment. ASCs, BM-MSCs, endothelial colony-forming cells (ECFCs), human placental amniotic membrane-derived MSCs (AMSCs), bone marrow-derived myeloid progenitor cells and bone marrow-derived CD34+ cells are all found to be potential cell choices for transplantation due to their optimal performance in pre-clinical studies.

##### ASCs

ERG demonstrates that ASCs injection can functionally improve PDR in oxygen-induced retinopathy (OIR) models, suggesting their potential use in PDR treatment [71]. Further studies indicated that ASCs can stabilize neovascularization by attaching maturing capillaries at pericyte positions and attenuating inflammatory microenvironment [43,71]. Several in vitro studies found that ASCs can exhibit therapeutic effect in PDR models potentially via juxtacrine interactions and extracellular vesicles [48,71]. In addition to all the findings above, however, ASCs cannot prevent neovascularization progression, which is the critical phenotype of PDR. Rather, neovascularization after the injection of ASCs is found increased by 54% in a study using OIR mice model, limiting the use of ASCs in PDR investigation [43].

##### Others

Several other subgroups of stem cells are also investigated as potential treatments of PDR. ECFCs injections are found to be effective in reducing the area of neovascularization without invasion into retina. Such injection can restore normal deep vascular plexus, with functioning connections between superficial and deep vascular plexus. The injected ECFCs remain in the vitreous body and eventually die without inflammation [72,73]. The therapeutic effect of ECFCs is further augmented via combination with bone-marrow derived CD34+ cells, as functional improvement can be detected after combinational injection, which was hardly detected after single ECFCs injection [72,73]. Several studies also found that BM-MSCs, AMSCs and bone marrow-derived myeloid progenitor cells are effective to reduce pathological neovascularization in PDR models [57,74,75]. However, more evidence is still required to understand the exact influence of these cells in pathological angiogenesis and their effect on PDR intervention.

### 4.2. Genetic-Based Therapy for DR

#### 4.2.1. Genetic-Based Therapy for NPDR

Treating NPDR with genetic approaches has become a novel research topic recently. Genetic therapies are interventions that aim at reaching therapeutic effect via editing specific gene expressions. For NPDR treatment, researchers focus more on protecting existing retinal vasculature and neurons from early-stage damage caused by hyperglycemia, leading to ERG improvement and functional preservation in diabetic retinopathy models [76,77].

##### RAS-Targeting Therapies

Many gene therapies are based on medications that have shown optimistic effects on NPDR such as those targeting the RAS system. Previous studies have shown the beneficial effects of angiotensin-converting enzyme inhibitors (ACEI) in treating NPDR [78]. However, the exact effect on NPDR using ACEI to suppress the RAS system is still under investigation. The delivery of angiotensin-converting enzyme 2 (ACE2) and angiotensin (1–7) into NPDR models can possibly demonstrate a similar protective effect on NPDR and serve as an alternative therapy [79,80]. Intravitreally adeno-associated virus (AAV) delivering ACE2 and angiotensin (1–7) into eNOS(−/−) diabetic rodents showed promising therapeutic effects such as decreased retinal vascular leakage, reduction in acellular capillaries, downregulation of inflammatory cells and oxidative stress [79]. Another study using AAV2-delivered ACE2 into STZ rats also demonstrated preventive and partially reversive results such as reduced acellular capillaries and inflammatory infiltration [80]. The research studies above suggest that genetically editing the ACE2/Ang (1–7) axis of RAS can protect the retina from hyperglycemia-induced retinopathies.

##### Anti-VEGF Therapies

Aside from the RAS system, the modification of existing therapeutic options is another method to select genetic targets. Treatment targeting VEGF can prevent NPDR progression to PDR. However, not all patients respond to anti-VEGF therapies [2]. The increased level of CTGF caused by anti-VEGF therapy is another adverse event to be considered, with the potential risk of developing fibrosis and tractional retinal detachment [33]. Genetic-based therapies are optimal to add on anti-VGEF therapies and amplify the therapeutic effects on NPDR. AAV-encoding angiostatin is found to be effective to reduce vascular leakage in STZ rodents via reducing diabetic-induced occludin loss, retinal VEGF increase and p42/p44 mitogen-activated protein (MAP) kinase phosphorylation [81]. Another study focusing on reducing CTGF expression using genetic therapy indicates that dual intervention with VEGF antibody and CTGF short hairpin RNA (shRNA) preserves retinal vascular ultrastructures better than either single-drug treatment [33]. Such studies suggest that genetic-based therapy can be used as a supportive intervention to improve NPDR outcomes.

##### Differentially Expressed Genes

Editing genetic expressions that are significantly changed is a promising way to develop novel treatments as well. Researchers have found elevated early growth response 1 (Egr1) and Nogo-B expression under hyperglycemic stress [76,82]. Therefore, these molecules may be potential targets for NPDR genetic treatment. Egr1 is a zinc finger transcription factor that can inhibit cell proliferation and elevate apoptosis level. Genetic intervention using sh-Erg1 can exhibit optimal effects both in vitro and in vivo. The overexpression of p53 reduced the therapeutic effect of sh-Egr1 in vitro, indicating that Egr1 mediates vasculature via the p53 pathway [76]. Nogo-B is a regulatory protein related to vascular homeostasis and remodeling, which is significantly elevated in patient samples and diabetic rats [82,83]. Nogo-B knockdown in diabetic rats significantly downregulated vascular leakage. However, it cannot be ignored that the knockdown of Nogo-B can result in an increased vascular permeability under normal conditions [82].

##### Neuroprotective Factors

Neuroprotective factors are becoming new research targets of NPDR gene therapy, since neural impairment is an important aspect of NPDR pathogenesis. The intraocular injection of AAV-encoded BDNF showed promising effect via upregulating BDNF level with increased living retinal ganglion cell number and improved function in STZ rats [84]. Erythropoietin (EPO) is a hematopoietic cytokine which demonstrated potential neuroprotective effects on DR. The subretinal injection of AAV2-cytomegalovirus (CMV)-EPO maintained BRB integrity and significantly reduced retinal cell apoptosis. Vision functions measured by ERG remain unchanged after one year of injection [85].

##### Oxidative Stress

As the central aspect of DR pathophysiology, oxidative stress is another optimal target for genetic adjustment. Researchers have found that epigenetic changes in manganese superoxide dismutase (MnSOD) are related to DR through various pathways, including metabolic memory phenomenon [86,87]. Therefore, the modulation of MnSOD is one of the upcoming NPDR genetic therapies under investigation. AAV-delivered MnSOD is found to be effective in preventing DR progression and development of the metabolic memory phenomenon. Pathological presentations such as retinal vascular basement membrane thickening, cell apoptosis and acellular capillaries are ameliorated after intravitreal administration of antioxidant gene therapy, offering a promising target for further investigation [88].

##### Diabetes

Genetic modification focusing on the pathophysiology of diabetes is a potential method to develop novel NPDR therapies on a more fundamental level. Urocortin 2 (UCN2) gene transfer focuses mainly on insulin sensitivity and availability. AAV8 encoded UCN2 can improve diabetes via increasing skeletal muscle glucose intake and insulin release. By treating diabetic conditions, transferring UCN2 can therefore improve ocular complications such as vascular leakage and retinal dysfunction as well, presenting as a potential genetic target for NPDR treatment [89]. 

##### Others

Other genetic targets including membrane attack complex (MAC) and microtubule associated protein 1 light chain 3 (LC3B) are also found effective to treat NPDR in pre-clinical studies. CD59 is one of the membrane-associated complement regulators, which is downregulated in DR patients and animal models. The AAV-encoded soluble membrane-independent form of CD59 (sCD59) can attenuate MAC deposition and hence reduce vascular and neuronal impairments such as vascular leakage, non-perfusion and retinal ganglion cell apoptosis [90]. Gene therapy targeting LC3B using anti-miR-204-5p showed vascular protective effect in DR models via the decreasing level of autophagy in SZT rats, which is also a potential therapy for further investigation [91].

#### 4.2.2. Genetic-Based Therapy for PDR

Pathological neovascularization is the typical presentation of PDR, which is also the key manifestation for PDR treatments to cope with. Genetic therapies targeting the pathophysiology of neovascularization, such as an imbalance of proangiogenic and antiangiogenic factors and endothelial proliferation disorder, are under investigation to determine their effect on PDR. 

##### Anti-VEGF Therapies

Anti-VEGF agencies are thoroughly investigated for their therapeutic effect on PDR as discussed above. However, due to the side effects of anti-VEGF injections, various gene-based therapies targeting VEGF are under investigation as potential alternative treatments for PDR. Soluble fms-like tyrosine kinase-1 (sFlt-1) is an antiangiogenic protein acting as an extracellular VEGF receptor, which can bind to VEGF and reduce its circulating concentration. By reducing VEGF level, AAV-delivered sFlt-1 can effectively downregulate vasculopathy and neovascularization in PDR rodents, supporting its optimal effect in PDR treatment [92,93,94,95,96]. Intracellular VGEF receptors such as Flt23k can also demonstrate a similar effect. One recent study demonstrated that self-complementary adeno-associated virus 2 (scAAV2)-encoded Flt23k can bind to intracellular VEGF to reduce retinal VEGF expression and neovascularization level in OIR rats via the administration of trimethoprim (TMP) as a stabilizer [97]. Due to the growth of bioengineering techniques, nanoparticles are also tested as novel choices to replace traditional virus vectors in genetic therapies, including those targeting PDR. A recent study using bioreducible lipidoid nanoparticles conveying VEGF small interfering RNAs (siRNAs) demonstrated optimal results in OIR rodents including reduced VEGF expression and neovascularization area. Histological changes of the VEGF siRNA-treated eyes are similar to those treated with ranibizumab, indicating the nanoparticles as successful vectors for siRNA to treat PDR [98].

##### Angiogenic Inhibitors

The genetic introduction of angiogenic inhibitors such as endostatin, angiostatin, pigment epithelium-derived factor (PEDF), calreticulin antiangiogenic domain (CAD), and tissue inhibitor metalloproteinase-3 (TIMP3) is also an effective way to treat PDR. Endostatin is an endogenous fragment of type XVIII collagen with antiangiogenic effects. The introduction of AAV-encoded endostatin is found to be effective in reducing neovascularization in OIR rodents, supporting its therapeutic effects in PDR [99,100]. Other attempts such as the regulated Müller cell delivery of endostatin and endostatin-modified EPCs can also demonstrate therapeutic benefits. However, these novel genetic modifications did not displace better therapeutic effects on pathological signs such as neovascularization and vascular leakage [101,102]. The modification of another antiangiogenic factor called angiostatin, a fragment of plasminogen, demonstrated a similar beneficial effect on OIR mice. Researchers have found that the injection of lentivirus-delivered angiostatin can successfully reduce neovascularization in 90% of the treated animals [103]. PEDF is a multifunctional protein with critical roles in many pathological processes. Researchers found that PEDF is related to angiogenesis and PEDF-modified PDR mice presented with decreased VEGF expression, inflammation and neovascularization level, offering evidence for clinical use of PEDF modification to treat PDR [104,105]. CAD is another antiangiogenic factor selected as genetic target for PDR therapy. Intravitreally AAV-delivered CAD can significantly reduce both choroidal and retinal neovascularization in a laser-induced and OIR model [106]. Preliminary studies of these angiogenic inhibitors all demonstrated optimal effects on PDR animal models. Therefore, further investigations are required to testify the safety and therapeutic effect of these interventions in larger samples.

##### Endothelial Proliferative Modulators

Endothelial proliferation is a crucial aspect of vascular formation. Therefore, endothelial proliferative modulators such as amino-terminal fragment (ATF), kringle1 domain of hepatocyte growth factor (HGFK1), or direct editing of endothelial cells are also potential options for PDR treatment. ATF is found to inhibit endothelial cell migration and therefore promote angiostasis in cancer models. Urokinase (uPA) and its receptor (uPAR) are crucial angiogenic factors impeding endothelial cell migration that connect with each other via light-chain fragment ATF. A study using AAV-delivered ATF modification in OIR rodents demonstrated reduced retinal neovascularization by 78.1%, but the safety of such an intervention is not reported [100]. HGFK was previously proved to inhibit endothelial cell proliferation and neovascularization in PDR models [107]. In vivo and in vitro studies both found that AAV-delivered HGFK1 is able to downregulate VEGF expression and normalize endothelial distribution, thus inhibiting pathological neovascularization in PDR models [108]. Furthermore, the direct genetic editing of endothelial cells to modulate its biological behavior is tested for its effects on PDR models. Studies have found that the direct knockdown of VEGF receptors using shRNA and clustered regularly interspaced short palindromic repeats (CRISPR) techniques are capable of reducing neovascularization in OIR rodents, indicating their optimal effects as future therapeutic choices [109,110].

### 4.3. Other Promising Strategies for DR

In addition to gene-based and cellular based resolutions, researchers are also investigating other fundamental therapies to cope with DR and improve treatment quality, such as novel drug delivery systems, epigenetic modulators, exosome-based treatments, natural products, etc. A novel intraocular drug delivery system can potentially avoid the side effect of repeated intravitreal injection. Therefore, materials such as polymeric molecules, nanoparticles, microparticles and intraocular devices such as ocular-implanted injection and sustained release drug delivery devices have been invented and clinically testified to optimize existing interventions [111,112]. Epigenetic modifications including DNA methylation, histone modification, non-coding RNA, and chromatin remodeling are also found involved in DR development, which offer novel biomarkers and treatment targets for researchers [113]. Epigenetic silencing of a diabetic-induced protein called thioredoxin-interacting protein is found effective in preventing DR progression via inhibiting the pathological neurovascular dysfunction both in vitro and in vivo [113]. Exosome intravitreal injection is also found to alleviate DR in diabetic rats, supporting the further investigation of exosome-based DR therapies [112]. Exosomes carrying non-coding RNA are also found crucial in diabetic vasculopathy [114]. Natural products including curcumin are promising ingredients for DR treatment as well. Research has shown that curcumin can protect retinal pigment epithelium (RPE) cells from the insults of high glucose via activating the Nrf-1/HO-1/ERK pathway, making it a potential therapeutic agent for DR [115]. Another study further supports the use of curcumin in treating DR and puts forward a potential protective mechanism of curcumin via reducing oxidative stress and maintaining the Nrf2 pathway homeostasis [116]. However, the exact mechanism of curcumin in treating DR is not fully understood yet, leaving a potential field for researchers to investigate [117]. These innovative interventions open up new directions for drug research and development.

## 5. Clinical Trials

Novel resolutions such as cellular and genetic based therapies are still emerging as new options for DR intervention, as animal studies are still proceeding. Therefore, only a few early-stage clinical trials have been assigned to investigate the effects of these methods. Due to the optimal evidence from animal studies, MSCs are selected as the potential cell type for DR trials (Table 1). A pilot study using autologous BM-MSCs transplantation to treat patients with both NPDR and PDR demonstrated positive results of reduced fasting blood glucose and C-reactive protein levels. However, only NPDR eyes demonstrated optimal effects at histological and functional levels, which were presented as reduced macular thickness and improvement of visual acuity [115]. In addition to this pilot study, multiple exploratory observational trials are also registered to test the efficiency and safety of human MSCs transplantation in treating DR patients (ChiCTR1800016870, IRCT201111291414N29). Phase 1 trials using CD34+ stem cells and UC-MSCs are recently registered to verify the safety of such transplantation in blinding diseases including DR (NCT01736059, NCT05147701). For genetic therapies, only VEGF-targeted transgenes are undergoing clinical trials for DR patients. Phase 2 trials using AAV-encoded gene therapies are registered to testify the efficacy, tolerability and safety of these interventions in DR patients with and without DME (NCT04567550, NCT04418427). Novel therapeutic targets such as epigenetic changes are still in the pre-clinical research stage, with no trials assessing their clinical application. Several epigenetic-targeting drugs are approved for leukemic diseases [116], but no epigenetic medication for DR is in clinical trials now. However, novel delivery systems including particle-based and implantation-based types are entering clinical practice (Table 2). Such trials opened a new field for DR interventions, which requires further trials and estimations to support the clinical use of these treatments. 

## 6. Conclusions and Future Directions

Compared to traditional treatment of DR, novel interventions concentrate on a more fundamental level of DR pathophysiology to treat diabetic insults. By focusing on retinal cellular and genetic performance, such novel interventions have limited systemic side effects and more focused therapeutic outcomes compared to conventional systemic medications. Long-term safety trials should be further conducted to testify these theories and support their clinical use. Furthermore, these novel interventions only require single injection, avoiding side effects of repeated intravitreal injections required by VEGF-targeting therapies. However, as DR is a part of systemic diabetes progression, controlling of primary disease should always be placed at the center of DR prevention and treatment. Localized novel interventions are the add-on methods to improve patients’ quality of life. Moreover, as the pathophysiology of DR varies from NPDR to PDR stage, cellular and genetic therapies that are beneficial for NPDR eyes may have the potential to aggravate PDR conditions due to their effect on promoting vascular growth. The diploma of vascular growth is the key problem of these novel therapies for researchers to understand and overcome. As cellular and genetic therapies are both novel techniques in treating DR with limited long-term study and clinical use, further research is required to better understand their therapeutic performance and long-term safety and stability in clinical practice. 

## Figures and Tables

**Table 1:** Novel stem cell-based trials for DR.

NCT Number	Study Title	Intervention	Condition or Disease	Phase	Status	First Posted
NCT01736059	Clinical Trial of Autologous Intravitreal Bone-marrow CD34+ Stem Cells for Retinopathy	CD34+ bone marrow stem cells intravitreal	Non-Exudative Age- Related Macular Degeneration Diabetic Retinopathy Retina Vein Occlusion Retinitis Pigmentosa Hereditary Macular Degeneration	Phase 1	Enrolling by invitation	2012
NCT05147701	Safety of Cultured Allogeneic Adult Umbilical Cord-Derived Mesenchymal Stem Cells for Eye Diseases	Biological: AlloRx	Eye Diseases Retinitis Pigmentosa Glaucoma Diabetic Retinopathy Macular Degeneration Traumatic Optic Neuropathy Optic Atrophy	Phase 1	Recruiting	2021
ChiCTR-ONC-16008055	Clinical study of mesenchymal stromal cells in treatment of diabetic retinopathy	Mesenchymal stem cells	Diabetic Retinopathy	Observational	Recruiting	2016
ChiCTR1800016870	A clinical study of human MSC-induced retinal pigment epithelium cells transplantation in the treatment of diabetic retinopathy	hMSC-RPE cells transplantation	Diabetic Retinopathy	Observational	Not Recruiting	2018
IRCT201111291414N29	Investigation on autologous mesenchymal stem cell transplantation in diabetic retinopathy	Autologous mesenchymal stem cells	Diabetic Retinopathy	Phase 0	Not Recruiting	2012

**Table 2:** Novel delivery system trials for DR treatment.

NCT Number	Study Title	Intervention	Ingredient	Condition or Disease	Phase	Status	First Posted
NCT00665106	Safety and Tolerability of NOVA63035 “Corticosteroid” in Patients With Diabetic Macular Edema Secondary to Diabetic Retinopathy	NOVA63035 (Dexamethasone prodrug emulsion)	Dexamethasone	Diabetic Macular Edema	Phase 1	Completed	2008
NCT00692614	A Study of MK0140 in Diabetic Patients With Macular Edema (0140-001)	I-vation triamcinolone acetonide implant (MK0140)	Triamcinolone acetonide	Macular Edema	Phase 2	Terminated	2008
NCT02424019	Phase 4 IOP Signals Associated With ILUVIEN^®^	ILUVIEN 0.19 MG	Fluocinolone acetonide	Diabetic Macular Edema	Phase 4	Completed	2015
NCT02399657	Effect of Dexamethasone Implant in Hard Exudate Complicated With Diabetic Macular Edema	Intravitreal dexamethasone 0.7 mg implant	Dexamethasone	Diabetes Mellitus Macular Edema Retinal Exudates and Deposits	Phase 4	Unknown	2015
NCT04503551	A Multicenter, Randomized Study in Participants With Diabetic Retinopathy Without Center-Involved Diabetic Macular Edema to Evaluate the Efficacy, Safety, and Pharmacokinetics of Ranibizumab Delivered Via the Port Delivery System Relative to the Comparator Arm	Port Delivery System with Ranibizumab (PDS) Implant	Ranibizumab	Diabetic Retinopathy	Phase 3	Active, not recruiting	2018
NCT03475407	The Effects of Intravitreal Ozurdex Implant in DME	Intravitreal Ozurdex implant	Dexamethasone	Diabetic Macular Edema	Phase 4	Unkown	2018
NCT04448496	Prospective Trial of Dexamethasone Implant for Treatment Naïve Diabetic Macular Edema	Dexamethasone implant	Dexamethasone	Diabetic Retinopathy	Phase 4	Recruiting	2020

## Data Availability

Not applicable.
